# Should azithromycin be used to treat COVID-19? A rapid review

**DOI:** 10.3399/bjgpopen20X101094

**Published:** 2020-05-13

**Authors:** Kome Gbinigie, Kerstin Frie

**Affiliations:** 1 GP and DPhil Student, Nuffield Department of Primary Care Health Sciences, University of Oxford, Oxford, UK; 2 Post-Doctoral Researcher, Nuffield Department of Primary Care Health Sciences, University of Oxford, Oxford, UK

**Keywords:** COVID-19, Azithromycin, Anti-Bacterial Agents, Primary health care, General practice

## Abstract

**Background:**

There are no established effective treatments for COVID-19. While novel drugs are being developed, azithromycin has been identified as a candidate treatment in the interim.

**Aim:**

To review the evidence for the effectiveness and safety of azithromycin in treating COVID-19.

**Design & setting:**

A rapid review of the literature was conducted.

**Method:**

Electronic searches were conducted on 16 April 2020 of PubMed, TRIP, EPPI COVID Living Map, MedRxiv, GoogleScholar, and Google. In vivo and in vitro studies were included assessing the safety and effectiveness of azithromycin for treatment of COVID-19, and/or the activity of azithromycin against SARS-CoV-2. In vivo studies needed to include a comparator group.

**Results:**

Three studies were identified, two in vitro and one in vivo, which were suitable for inclusion. All three were published as pre-prints. The in vitro studies revealed conflicting results, with one finding anti-SARS-CoV-2 activity for azithromycin alone, while the other found activity against SARS-CoV-2 only when azithromycin was combined with hydroxychloroquine. A small trial of 36 patients, with high risk of bias, found superior viral clearance in patients with COVID-19 treated with azithromycin and hydroxychloroquine combined, compared with hydroxychloroquine alone.

**Conclusion:**

There is no evidence to support the use of azithromycin for the treatment of COVID-19 outside of the context of clinical trials, unless it is used to treat bacterial super-infection. There is extremely limited evidence of a possible synergy between azithromycin and hydroxychloroquine. The adverse events profile of azithromycin in the context of COVID-19 has not yet been established. Well-conducted clinical trials are urgently needed in this area.

## How this fits in

There are no treatments with proven effectiveness for COVID-19, and there has therefore been interest in re-purposing existing medications in the current pandemic. Azithromycin is being used widely off-label to treat COVID-19. This review did not identify any evidence to support the use of azithromycin alone for the treatment of COVID-19, in the absence of evidence of bacterial super-infection. There is extremely limited evidence, with high risk of bias, to suggest that azithromycin may have a synergistic effect when combined with hydroxychloroquine.

## Introduction

COVID-19 was declared a pandemic by the World Health Organization on 11 March 2020.^1^ Intensive work is being conducted internationally to develop novel treatments for, or a vaccine to prevent, COVID-19. In the interim, there have been attempts to re-position a number of existing medications to treat COVID-19.

One candidate is azithromycin, a macrolide antibiotic predominantly used to treat respiratory, skin, and soft-tissue infections.^2^ There is some in vitro evidence that azithromycin may prevent replication of other viruses, such as human influenza virus H1N1^3^ and Zika virus.^4^ The possible mechanism of action of azithromycin against SARS-CoV-2 is currently unknown, however some theories have been postulated. Poschet and colleagues^5^ found in an in vitro study that azithromycin led to an increase in the pH of host cells, which may impede viral entry, replication, and spread. Moreover, SARS-CoV-2 is believed to possess a unique furin-like cleavage site in the spike protein^6^ (the protein that facilitates viral entry into host cells). Poschet *et al* found that azithromycin reduces levels of the enzyme furin in host cells, and may therefore interfere with viral entry. Furthermore, macrolide antibiotics are reported to reduce the production of pro-inflammatory cytokines,^5,7^ which may abate the pro-inflammatory state induced by SARS-CoV-2 infection.

Azithromycin is being used internationally off-label to treat patients with COVID-19.^8^ An online survey of 6227 physicians in 30 countries at the end of March 2020^9^ found that azithromycin was the second most commonly prescribed treatment for COVID-19 after simple analgesia.^10^ Forty-one per cent reported that they had personally prescribed azithromycin, or had seen it prescribed for COVID-19, and it was rated as the second most effective treatment for COVID-19 after hydroxychloroquine/chloroquine.^10^

In light of these striking figures, it is of critical importance to evaluate the evidence for the effectiveness of azithromycin in the current pandemic. Moreover, it is vital to assess its safety profile in the context of COVID-19. Side effects of azithromycin for its present indications commonly include gastrointestinal upset,^2^ and uncommonly, prolongation of the QT interval. Caution must therefore be applied if azithromycin is prescribed to patients who might be predisposed to QT interval prolongation.^2^ Azithromycin should be used with caution or avoided in patients with severe hepatic or renal failure.^2^

The aim of this rapid review is to determine the effectiveness and safety of azithromycin for the treatment of COVID-19.

## Method

Electronic searches were conducted of PubMed, TRIP, EPPI COVID Living Map, MedRxiv, GoogleScholar, and Google on 16 April 2020 (see Appendix S1 for search strategy). In keeping with a pragmatic rapid review, the research team stopped screening Google search results when a page was reached with no relevant links. In vitro studies were included assessing the activity of azithromycin against SARS-CoV-2. In vivo studies were included assessing the effectiveness and/or safety of azithromycin for the treatment of COVID-19. Patients of all ages and sexes were included. The intervention was defined as the treatment of COVID-19 with azithromycin, and for inclusion, in vivo studies needed to provide data allowing comparisons to be made between patients who did and did not receive azithromycin. The comparator was any other treatment, or no treatment. Case-reports and studies that did not use a comparative design were therefore excluded. Systematic reviews were also not included, but were used as a point of reference. Studies needed to report outcomes relating to effectiveness and/or safety, but no follow-up time to assess the outcome was specified. No language restrictions were imposed, but studies published before 2019 were excluded. The authors independently screened titles and abstracts for separate sets of databases. Following full-text screening, one author extracted in vivo data and the other in vitro data; both authors checked the data extracted by the other. Demographic data, information on interventions used, and outcome data were extracted from included in vivo studies. The Cochrane criteria^11^ were used to assess the quality of included in vivo studies. The authors independently performed a risk of bias assessment, with disagreement resolved through discussion. This article presents a narrative summary of the identified studies.

## Results

**Figure 1.**
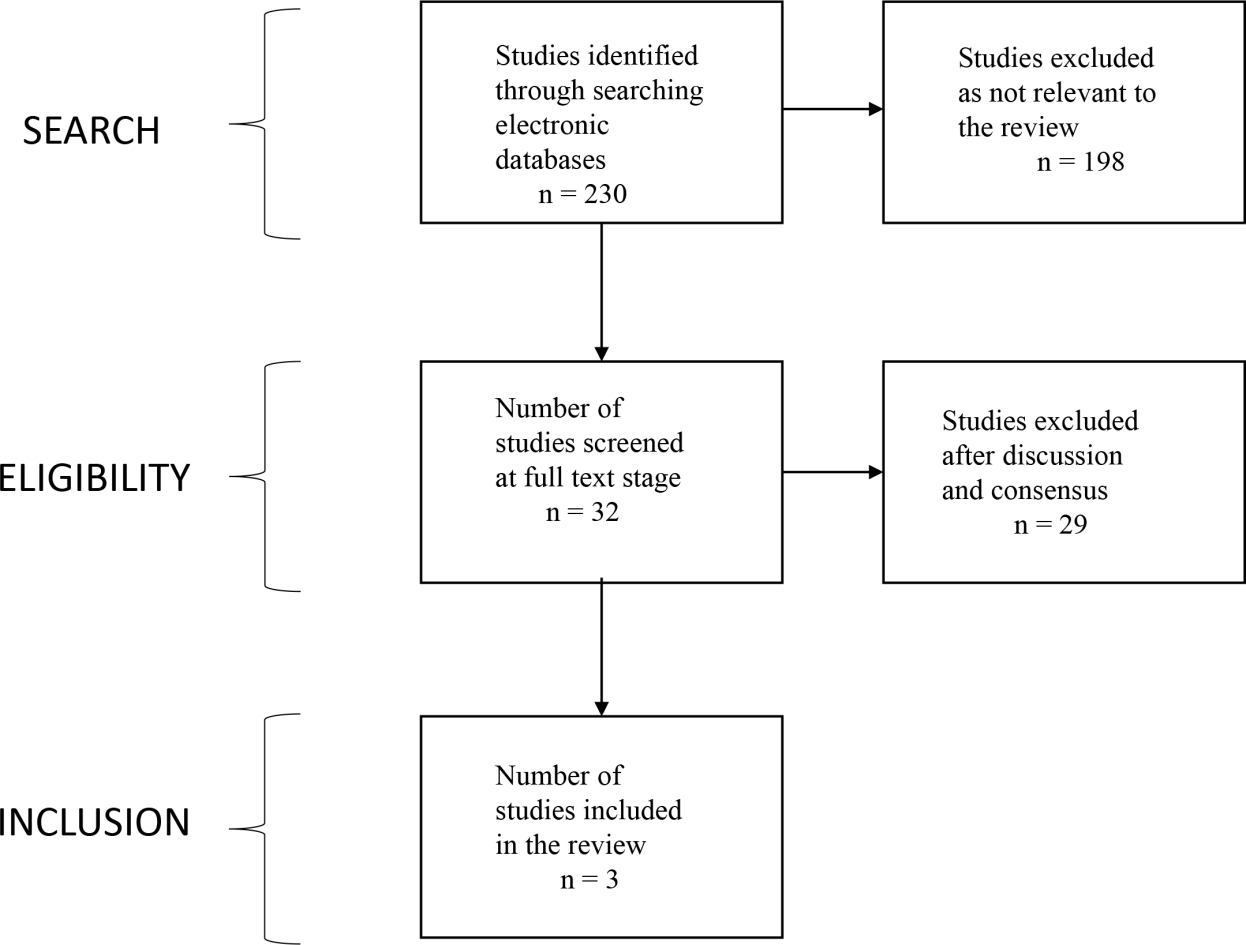
Flowchart showing the process for identification of studies suitable for inclusion

Searches of electronic databases yielded 230 articles (excluding Google). The first 140 Google hits were screened. Thirty-two eligible articles were identified. Of these, three studies, two in vitro and one in vivo, were suitable for inclusion (see Figure 1 ).

### In vitro research

In a pre-print, Touret *et al* ^12^ assessed the in vitro ability of 1520 drugs to inhibit SARS-CoV-2 activity at a MOI of 0.002 (multiplicity of infection; that is, the ratio of virions to host cells). Azithromycin was found to have an EC50 (half-maximal effective concentration; that is, the concentration at which viral RNA increase is inhibited by 50%) of 2.12 µM; a 50% cytotoxic concentration (CC50; that is, the concentration that results in 50% cell death) of >40 µM; and a selectivity index of >19. The authors therefore concluded that, among other drugs, azithromycin could be used in the treatment of COVID-19.

In another pre-print, Andreania and colleagues^13^ report the results of an in vitro study assessing the activity of azithromycin and hydroxychloroquine against SARS-CoV-2. They tested azithromycin at concentrations of 2, 5, and 10 µM against the virus. At both a low and high MOI (0.25 and 2.5, respectively), azithromycin alone did not inhibit viral replication. However, at a low MOI and when azithromycin 5 or 10 µM was combined with hydroxychloroquine 5 µM, the authors reported that viral replication was inhibited. At a high MOI, they found that azithromycin 10 µM combined with hydroxychloroquine 2 µM inhibited viral replication. The authors reported that the concentrations of both drugs used in this study reflected those achieved in lung tissue in vivo; the findings may therefore better reflect what might happen in the human body.

The MOI in the two in vitro studies was very different, and was lower by a factor of 100 in the study by Touret *et al*.^12^ This may be a contributory factor to Touret and colleagues finding that azithromycin alone had activity against SARS-CoV-2, while Andreania *et al* did not.

### In vivo research

#### ​Effectiveness

Only one trial was identified on the effectiveness of azithromycin for the treatment of COVID-19, conducted by Gautret and colleagues in France and reported in a pre-print^14^ (see Table 1). The researchers recruited 20 patients to their trial investigating the effectiveness of hydroxychloroquine (200 mg ter die sumendum for 10 days), and compared clinical outcomes against 16 control cases. Of the twenty patients who received hydroxychloroquine, six additionally received azithromycin to prevent super-infection (500 mg on Day 1, then 250 mg daily for 4 days). Patients receiving azithromycin had a daily electrocardiogram (ECG) to monitor their QT interval. The authors found that the six patients who received azithromycin combined with hydroxychloroquine were significantly more likely to test negative for SARS-CoV-2 on Days 3, 4, 5, and 6. On Day 6, 57.1% of the hydroxychloroquine group, compared to 100% of the combined hydroxychloroquine and azithromycin group, were virologically cured (*P*<0.001). The researchers argue that this suggests that there is a synergistic effect of azithromycin combined with hydroxychloroquine for the treatment of COVID-19.

**Table 1.** Summary of characteristics of identified in vivo study

**Authors (Year**)	**Country**	**Setting**	**Sample size (treatment/control**)	**Mean age (SD**)	**Inclusion criteria**	**Treatment**	**Primary** **outcome**	**Findings**
Gautret *et al* (2020)	France	Hospitals in Marseille, Nice, Avignon and Briançon	6 – AZT and HCQ14 – HCQ alone16 – control	Treatment groups: 51.2 (18.7)Control: 37.3 (24.0)	SARS-CoV-2 carriage in nasopharyngeal sample Age>12 years (treatment groups only)	Six patients received 500 mg AZT on Day 1 then 250 mg daily for four days and200 mg HCQ three times a day for 10 days.14 patients received 200 mg HCQ three times a day for 10 days.	Outcome of a nasopharyngeal swab on Day 6	Virological cure:AZT and HCQ – 100%HCQ alone – 57.1%Controls – 12.5% p<0.001

AZT = azithromycin; HCQ = hydroxychloroquine.

It is important to highlight several limitations of the trial by Gautret and colleagues, which have also been discussed elsewhere.^15^ The study had a high risk of bias (see Table 2). The sample size was small, meaning that the analysis was likely underpowered, which can lead to false positive results.^16^ The authors state that the decision to treat patients with azithromycin was based on clinical presentation. However, they do not clarify the criteria used to make this decision, making it difficult to assess the potential of selection biases. Furthermore, one of the six patients in the combined treatment group who tested negative for SARS-CoV-2 on Day 6 subsequently tested positive on Day 8. This indicates possible fallibility of the test, and demonstrates the need for longer-term follow-up data. In addition, the trial did not include a treatment arm of azithromycin alone. The trial therefore does not provide evidence for the effectiveness of azithromycin on its own, and any possible effects may be dependent on co-administration with hydroxychloroquine.

**Table 2.** Risk of bias assessment for the included in vivo study

**Bias**	**Rapid review authors’ judgment**	**Reason for judgment**
Random sequence generation	High risk	Non-randomised trial
Allocation concealment	High risk	Open-label
Blinding	High risk	Open-label
Incomplete outcome data	High risk	Intention-to-treat analysis not performed
Selective reporting	Unclear risk	No adverse event data reported, although the authors state that this will be reported separately at the end of the trial
Other bias	High risk	Under-powered according to the authors’ own power calculation

#### ​Safety data

The authors did not identify any comparative studies assessing the safety profile of azithromycin in the context of COVID-19. The trial by Gautret *et al* did not report any adverse events, although they did state that a further paper would address any side effects identified during the trial.

## Discussion

Only three studies were identified that were suitable for inclusion. The in vitro studies provided mixed results, with one study finding a significant effect of azithromycin against SARS-CoV-2, while the other found significant inhibition of viral replication only when azithromycin was combined with hydroxychloroquine. Only one comparative trial was identified assessing azithromycin treatment for COVID-19 in vivo. The trial, which included a small number of participants, found that azithromycin combined with hydroxychloroquine led to a significant improvement in viral clearance compared to hydroxychloroquine alone. Due to considerable methodological limitations of the trial, there is a high risk of bias. No safety data were reported.

### Comparison with other literature

The same research team that conducted the in vivo study included in this review conducted a single-arm trial of 80 patients who tested positive for SARS-CoV-2 and showed mild symptoms,^17^ to further assess the effectiveness of the combined hydroxychloroquine/azithromycin treatment regime. Their sample included the six patients of the in vivo study discussed in this review who received both azithromycin and hydroxychloroquine.^14^ Patients had a baseline and Day 2 ECG to assess the QT interval. The authors report that patients experienced only few and minor side effects, including two cases of nausea, four of diarrhoea, and one of blurred vision. They found that over 80% of patients tested negative for the virus on Day 7, and over 90% tested negative on Day 8. These results are thus in keeping with the reported synergy between azithromycin and hydroxychloroquine found by the three-arm trial by Gautret *et al*,^14^ as well as one in vitro study.^13^ However, the sample size was small and there was no control group.

The same group of researchers also report the results of a non-comparative observational study.^18^ The research team used the same combination of azithromycin and hydroxychloroquine outlined above, but this time assessed outcomes over at least 9 days in 1061 patients, finding that over 90% of patients were virologically cured on Day 10, and over 95% on Day 15. Eight patients (0.75%) died and 41 required further treatment. The authors report that the mortality in their cohort was significantly lower than that of 720 patients treated with other drugs in hospitals of the same region (mortality rate of 6.5%). The study thus provides some evidence for the effectiveness of the combined treatment.

By contrast, Molina and colleagues,^19^ who treated 11 patients with the same combined treatment protocol described by Gautret and colleagues, found poor outcomes in nine of these patients. One patient died, and only two tested negative for SARS-CoV-2 on Day 6. While the study was underpowered, these findings cast some doubt on the effectiveness of the treatment regime.

The lack of a control group in these three studies makes it difficult to interpret the results, as the influence of confounding factors that may have influenced the patients’ outcomes cannot be ruled out.^20^ The cohort analysis of 1061 patients compared the mortality rates of patients receiving combined azithromycin/hydroxychloroquine treatment, with those not receiving this combination. However, such non-randomised designs are more likely to introduce allocation bias.

No studies were found assessing the safety of azithromycin treatment in the context of COVID-19, but a multi-centre self-controlled case series study was identified, which assessed primary and secondary care medical record data between 2000 and 2020 for 956 374 patients in six countries.^21^ The authors of that study identified a significantly increased risk in cardiovascular mortality, chest pain, and heart failure in patients who were treated with azithromycin and hydroxychloroquine compared to hydroxychloroquine alone. While the findings are not specific to the context of COVID-19 disease, these results indicate that adverse cardiovascular events are more likely to occur when hydroxychloroquine and azithromycin are combined.

## Strengths and limitations

The present authors kept the search criteria deliberately broad to maximise the chance of capturing eligible studies. Both in vitro and in vivo studies were included. Given the importance of the review topic and the need to disseminate the findings in a timely fashion, the review was conducted within a week. The authors employed methods closely aligned with that of a systematic review.

However, given the rapid pace of publications on COVID-19, it is possible that the authors have not identified all studies suitable for inclusion, particularly unpublished and non-English language studies. They tried to mitigate this by searching multiple sources, including Google and pre-print servers. Furthermore, no language restrictions were applied.

The authors also recognise the significant limitations of the included studies. All three studies were published as pre-prints and have therefore not yet been accepted for publication through the peer review process. Furthermore, no trial was identified comparing the effectiveness of azithromycin alone against a control group; inferences concerning the effectiveness of azithromycin as a standalone treatment cannot, therefore, be made. The in vivo study was methodologically flawed, and there were inconsistencies in the findings of the in vitro studies.

### Implications for research and practice

There is a dearth of evidence for prescribing azithromycin alone or in conjunction with hydroxychloroquine for COVID-19. Additionally, existing studies have severe methodological limitations. As a result, the present authors cannot currently recommend the use of azithromycin for COVID-19, outside the context of research studies. It is recognised, however, that some clinicians may wish to prescribe azithromycin to treat a suspected bacterial pneumonia that has complicated COVID-19, particularly if this forms part of their local or national treatment guidelines for pneumonia. The authors have not identified convincing evidence to support the co-prescription of azithromycin and hydroxychloroquine, and would advise extreme caution in adopting this approach. Both hydroxychloroquine and azithromycin can prolong the QT interval, putting susceptible patients at increased risk of *torsade de pointes*. For this reason, a number of international guidelines recommend daily ECGs when these drugs are combined.^22^ This is particularly important for patients in primary care, for whom it is not usually feasible to perform regular ECGs.

Using drugs with unproven effectiveness during pandemics can lead to unintended consequences and harms.^23^ For instance, widespread azithromycin prescription in the community may cause rising levels of antimicrobial resistance, a harm that is unlikely to be captured in clinical trial reporting. In light of this, and the fact that physicians worldwide are using azithromycin off-label to treat COVID-19, there is an urgent need for well-conducted, adequately powered, ideally double-blinded randomised clinical trials in this area, with careful reporting of adverse events. Several trials comparing azithromycin/hydroxychloroquine to hydroxychloroquine alone have been registered on the ClinicalTrials.gov. The results of these studies will hopefully guide clinical practice during the present pandemic, including defining an optimal dosing regimen.

The authors did not identify any results of trials assessing the use of azithromycin as a standalone treatment. They did, however, identify the registration of a placebo-controlled randomised clinical trial of azithromycin in the primary care setting,^24^ the results of which should help provide safety and effectiveness data in the outpatient setting.

No in vivo studies were identified assessing the safety or effectiveness of azithromycin as a standalone treatment for COVID-19. There is very limited in vitro and in vivo data suggesting a possible synergy of azithromycin and hydroxychloroquine. Only one comparative in vivo study was identified, with a small number of patients and considerable methodological limitations. At present, there is insufficient evidence to support the safety and/or effectiveness of azithromycin alone, nor in combination with hydroxychloroquine, for the treatment of COVID-19.
